# Spontaneous Cancer-Stromal Cell Fusion as a Mechanism of Prostate Cancer Androgen-Independent Progression

**DOI:** 10.1371/journal.pone.0042653

**Published:** 2012-08-03

**Authors:** Ruoxiang Wang, Xiaojuan Sun, Christopher Y. Wang, Peizhen Hu, Chia-Yi Chu, Shurong Liu, Haiyen E. Zhau, Leland W. K. Chung

**Affiliations:** 1 Uro-Oncology Research, Department of Medicine, Cedars-Sinai Medical Center, Los Angeles, California, United States of America; 2 Molecular Urology and Therapeutics, Department of Urology, Emory University School of Medicine, Atlanta, Georgia, United States of America; Innsbruck Medical University, Austria

## Abstract

We have previously shown that human prostate cancer cells are capable of acquiring malignant attributes through interaction with stromal cells in the tumor microenvironment, while the interacting stromal cells can also become affected with both phenotypic and genotypic alterations. This study used a co-culture model to investigate the mechanism underlying the co-evolution of cancer and stromal cells. Red fluorescent androgen-dependent LNCaP prostate cancer cells were cultured with a matched pair of normal and cancer-associated prostate myofibroblast cells to simulate cancer-stromal interaction, and cellular changes in the co-culture were documented by tracking the red fluorescence. We found frequent spontaneous fusions between cancer and stromal cells throughout the co-culture. In colony formation assays assessing the fate of the hybrid cells, most of the cancer-stromal fusion hybrids remained growth-arrested and eventually perished. However, some of the hybrids survived to form colonies from the co-culture with cancer-associated stromal cells. These derivative clones showed genomic alterations together with androgen-independent phenotype. The results from this study reveal that prostate cancer cells are fusogenic, and cancer-stromal interaction can lead to spontaneous fusion between the two cell types. While a cancer-stromal fusion strategy may allow the stromal compartment to annihilate invading cancer cells, certain cancer-stromal hybrids with increased survival capability may escape annihilation to form a derivative cancer cell population with an altered genotype and increased malignancy. Cancer-stromal fusion thus lays a foundation for an incessant co-evolution between cancer and the cancer-associated stromal cells in the tumor microenvironment.

## Introduction

Prostate cancer treatment is frequently set back by androgen-independent progression and bone metastasis. Whereas primary cancer is initially androgen-dependent and may be curable by androgen deprivation, androgen-independence is common to recurrent cancer and metastasis, which are often incurable. Along with progression from the primary to the metastatic state, cancer cells have acquired certain traits favorable for survival in the absence of androgens [1], [2].

The cause of androgen independence remains to be elucidated. Elevated androgen receptor (AR) activity and enhanced survival in the cancer cells may be contributing factors [3], [4], but stromal cells in the tumor microenvironment also play an important role [5]. In normal prostate, the glandular epithelial layer is structurally separated from the surrounding stroma by a laminar basement membrane. In prostate cancer, infiltrating cancer cells would form direct contacts with the stromal cells, placing the cancer progression process in the context of a stromal microenvironment. Delineating the mechanism of cancer-stromal interaction is a priority for improvement of prostate cancer treatment.

We have defined an obligatory role of the mesenchymal stroma in prostate cancer progression to androgen independence by modeling the interaction between cancer and stromal cells [6], [7], [8], [9]. LNCaP human prostate cancer cells, for instance, are androgen-dependent when assayed for tumor formation in castrated male athymic mice. These cells, however, could form frequent tumors when co-inoculated with cells of the bone marrow mesenchymal stromal lineage [9], [10]. Intriguingly, cancer cells recovered from the resultant tumors were androgen-independent, constitutively producing high levels of Prostate Specific Antigen (PSA), reproducibly forming androgen-independent xenograft tumors, and frequently showing metastatic capability to bone [9], [10]. To simulate the *in vivo* cancer-stromal interaction, we co-cultured the cancer and stromal cells under conventional and 3-dimensional conditions. Upon direct contact, the myofibroblast stromal cells could rescue LNCaP cells from androgen starvation-induced death [11], while 3-dimensional co-culture resulted in constitutive expressional changes in both the cancer and the stromal cells [12], [13], reflecting the co-evolution between cancer and mesenchymal stromal cells observed in prostate cancer progression and bone metastasis. Intriguingly, cancer cells retrieved from the co-culture bore permanent genomic alterations, detected by the appearance of marker chromosomes. Genomic alteration may be the foundation for aneuploidy, the most conspicuous abnormality in metastatic cancers [14], [15]. Investigation into the direct contact between cancer and stromal cells may unveil the mechanism by which cancer-stromal interaction promotes prostate cancer progression and bone metastasis.

In this report, we employed co-culture methods to further investigate the cause of androgen independence. LNCaP cells tagged with a red fluorescence protein were overlaid onto a monolayer of prostate myofibroblast cells to facilitate direct contact between the two cell types. By tracking the red fluorescence, we found that cancer cells could spontaneously fuse with stromal cells. By following the fate of the cancer-stromal hybrids, we found that most of the fused cells died, while a few could survive to form clones. The derivative clones exhibited chromosomal loss, with accelerated growth and elevated PSA production in an androgen-independent manner. Cancer-stromal cell fusion is thus a contributing mechanism for androgen-independent prostate cancer progression.

## Methods

### Cells and cell culture conditions

The origin of the LNCaP human prostate cancer cell line used in this study was previously reported [16]. The establishment of RL-1, a LNCaP clone expressing an AsRed2 fluorescence protein, together with the isolation and characterization of a matched pair of HPS-14 normal and HPS-15 cancer-associated human prostate myofibroblast stromal clones was previously reported [11]. The MRC-5 and the MRC-9 fetal human lung stromal cell lines were purchased from American Type Culture Collection (Manassas, VA). PrSC, a primary normal human prostate stromal cell line, was purchased from Lonza Walkersville, Inc. (Walkersville, MD). All these cells were maintained at 37°C in T-medium (Invitrogen, Carlsbad, CA) containing 10% fetal bovine serum (FBS), ampicillin (100 units/ml) and streptomycin (100 µg/ml), with humidified air supplemented with 5% CO_2_. Normal human bone marrow multipotent mesenchymal stromal cells transduced with a lentiviral green fluorescence protein (hMSC-GFP) were purchased from the Tulane Center for Gene Therapy (Tulane University, New Orleans, LA) and cultured in α Minimum Essential Medium (Invitrogen) containing 16.5% FBS and 2 mM L-glutamine [17].

### Co-culture

Direct co-culture of the cancer and stromal cells was previously reported [11]. To form a stromal cell monolayer, 5×10^5^ stromal cells in 2 ml medium were plated in each well of a 6-well plate, and allowed to grow into full confluence. The medium was removed and 5×10^5^ cells of the red fluorescent LNCaP RL-1 clone in 4 ml fresh medium were overlaid onto the monolayer. The co-culture medium was changed every three days.

### Colony formation assay

Cells being studied were collected following trypsin-EDTA treatment and diluted to a low density single-cell suspension (4 cells/ml). To each well in a 96-well plate, 100 µl of the suspension was applied. After 24 hours of incubation, the plates were examined under microscope and wells containing a single cell were marked. After 8 weeks of culture, colonies formed in the marked wells were amplified for further characterization.

### Androgen treatment

The protocol for treating cultured cells with androgen was previously reported [18]. Briefly, equal numbers of cells (5×10^5^/well) were plated onto 6-well plates. After attachment, androgen-deprivation was done by culturing the cells with phenol red-free RPMI 1640 medium (Invitrogen) for 48 hours. The medium was changed to phenol red-free RPMI 1640 containing 1% dextran-charcoal stripped FBS. Synthetic androgen methyltrienolone (R1881, Perkin Elmer, Waltham, MA) was added to the treatment group to 5 nM. After 24 hours of treatment, the culture medium was collected for PSA measurement and the cells were collected for whole cell lysate preparation.

### PSA ELISA

Cell culture medium was used to detect PSA production using our previously reported protocol [11]. A commercial PSA ELISA kit (United Biotech Inc., Mountain View, CA) was used and triplicate assays were performed on each sample.

### Cell proliferation assay

The protocol used for assaying cell proliferation with MTT conversion was reported previously [11]. Briefly, equal numbers of cells (5×10^5^/ml) were plated onto a 96-well plate. After androgen treatment as described above, the cells were subjected to a proliferation assay. Triplicate assays were performed on each group.

### Western blotting

The protocol for Western blotting was reported previously [18]. In this study, 20 µg of whole cell lysate protein was fractionated and blotted onto nitrocellulose membrane. The membrane was detected with specific antibodies to AR (Santa Cruz Biotechnologies, Santa Cruz, CA). The level of the β-actin was used as loading control.

### PCR analysis

The conditions for genomic DNA amplification were reported previously [18]. In this study, sex chromosomes were detected with the forensic sexing method [19]. The primer pair used for detection of amelogenin genes was 5′- CTGATGGTTGGCCTCAAGCCTGTG-3′ and 5′- TAAAGAGATTCATTAACTTGACTG-3′ [20]. This pair detects both the X-linked and Y-linked amelogenin genes, producing a 977 base pair (bp) product from intron 3 of the X-linked gene and a 788 bp product of the Y-linked gene in a single PCR analysis. A primer pair of 5′-ATGCAATCATATGCTTCTGCTATGTTAAGC-3′ and 5′-CTACAGCTTTGTCCAGTGGCTGTAGCGGTC-3′ was used to detect the coding region (615 bp) of the Y-specific SRY gene. The product of the reaction was fractionated by 1% agarose gel electrophoresis and visualized by ethidium bromide staining.

### Fluorescence-activated cell sorting (FACS) analysis

The protocol for the FACS assay was described previously [11]. In this study, cultured cells were detached by trypsin-EDTA. After being washed in phosphate buffered saline and fixed in 75% ethyl alcohol, the cells were stained with propidium iodide in the presence of RNase A for 30 minutes and subjected to FACS analysis for cell cycle profiling. Peripheral blood mononuclear cells from two healthy males were used as normal control for genomic content. Use of donor sample was approved by an Institutional Review Board of the Emory University School of Medicine (IRB number 278-2006), with written informed consent from the participants.

### Fluorescence microscopy

The protocol for red fluorescence imaging was previously reported [11]. In this study, for comparison purposes all the red fluorescent images were taken with fixed settings: 4.15 seconds for imaging at 40× magnification, 2.075 seconds for imaging at 100× magnification, and 1.0375 seconds for imaging at 200× magnification.

## Results

### 1. Characteristics of the prostate cancer and stromal cells in the co-culture system

**Figure 1:**
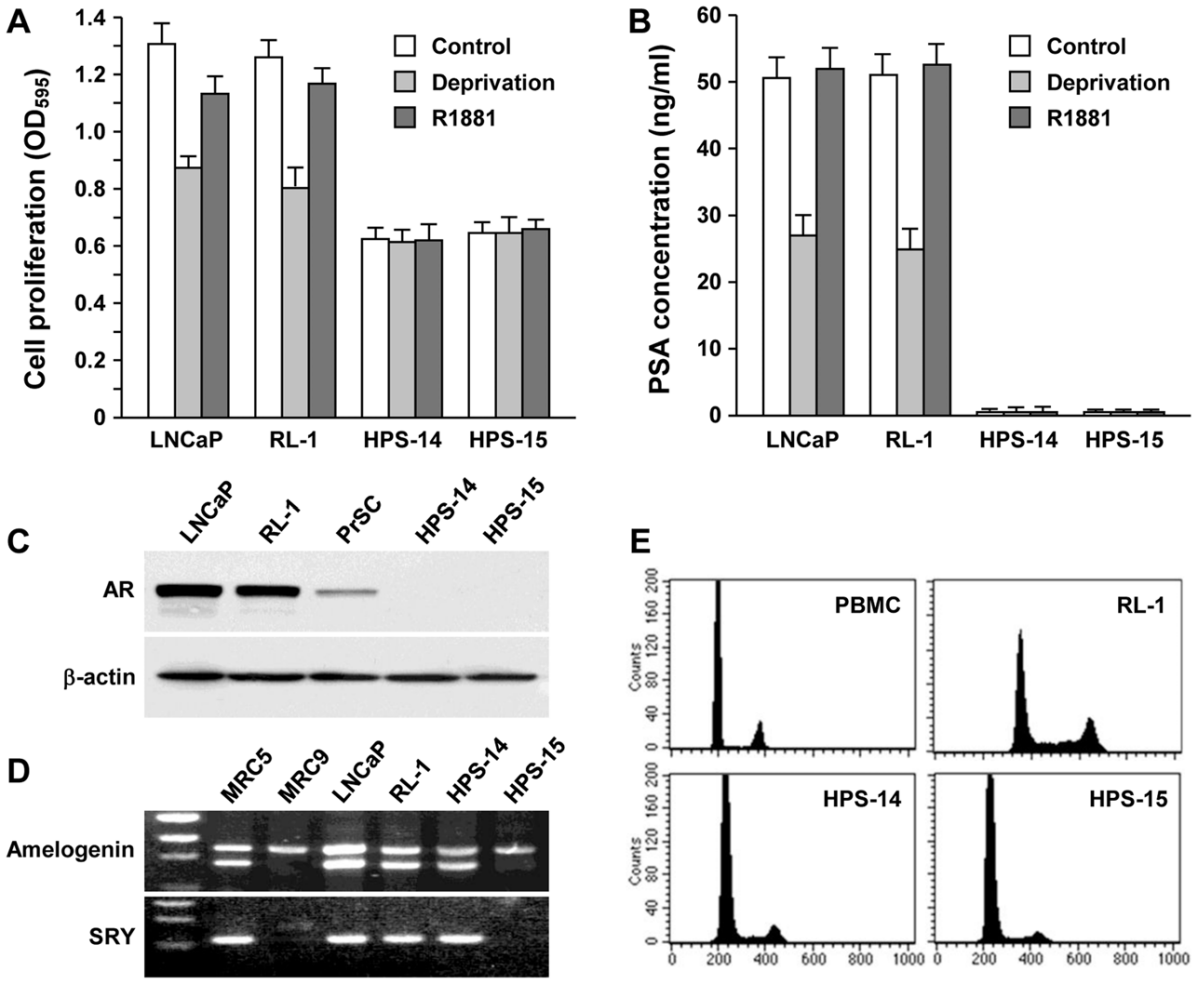
Differential characteristics of cells used for cancer-stromal co-culture. **A,** Differential growth response to androgen. The original LNCaP cells and the red fluorescent RL-1 clone, together with a matched pair of prostate stromal cells, HPS-14 (normal) and HPS-15 (cancer-associated), were compared for proliferation by MTT conversion. After 48-hour serum starvation, the cells were treated in regular culture medium (Control), androgen deprivation medium (Deprivation), and androgen deprivation medium containing 5 nM synthetic androgen R1881 (R1881). MTT assays were conducted 48 hours later. Data represent the mean±SD of a triplicate assay. **B,** Differential PSA production. Culture medium from the treated cells was detected for PSA concentration with ELISA. Each data point represents the mean±SD of triplicate assays. **C,** Differential AR expression. Cells were subjected to Western blotting for AR expression. The normal human stromal PrSC cell line was used in the comparison. The level of β-actin expression was used as a loading control. The result was representative of two separate experiments. **D,** Differential karyotype. Genomic DNA PCR was used to detect sex chromosomes with a forensic sexing method [20]. MRC-5 and MRC-9 cells were to prepare male and female genomic DNA, respectively. In the upper panel (Amelogenin), X chromosome was detected as the upper band, while Y chromosome was detected as the lower band. In the lower panel (SRY), chromosome-specific SRY locus was used to confirm the presence of Y chromosome. **E,** Differential genomic contents. Peripheral blood mononuclear cells (PBMC) from a healthy male were used to show normal diploid genomic content as measured by FACS. RL-1 cells had a near-tetraploid genomic content, similar to the parental LNCaP cells [21]. HPS-14 and HPS-15 cells showed genomic contents similar to the normal PBMC.

We used a red fluorescence protein to track the LNCaP cells in co-culture [11]. A representative red fluorescent LNCaP clone, RL-1, was used in this study. RL-1 cells displayed growth rates (Figure 1A), PSA production (Figure 1B) and AR level (Figure 1C) similar to the parental LNCaP cells. At the genomic level, RL-1 cells contained the Y chromosome (Figure 1D), a genomic feature that was lost upon androgen-independent progression [21]. Both RL-1 and the parental LNCaP cells displayed an identical near-tetraploid genomic content (Figure 1E), indicating an XXYY karyotype [21]. When assayed for xenograft tumor formation, RL-1 cells did not form subcutaneous tumors in athymic mice, thus retaining the non-tumorigenic property of the parental LNCaP cells.

We previously characterized matched pairs of normal HPS-14 and cancer-associated HPS-15 human prostate stromal clones [11], which were large and slow-growing myofibroblasts (Figure 1A) not expressing PSA (Figure 1B). Interestingly, neither of the stromal clones expressed detectable levels of AR by Western blotting (Figure 1C) or by RT-PCR (data not shown). HPS-14 cells contained the Y chromosome, which was lost in the cancer-associated counterpart (Figure 1D). Flow cytometry revealed that both HPS-14 and HPS-15 maintained a genomic content close to that of normal human peripheral blood mononuclear cells (Figure 1E).

### 2. Spontaneous fusion of cancer and stromal cells

**Figure 2:**
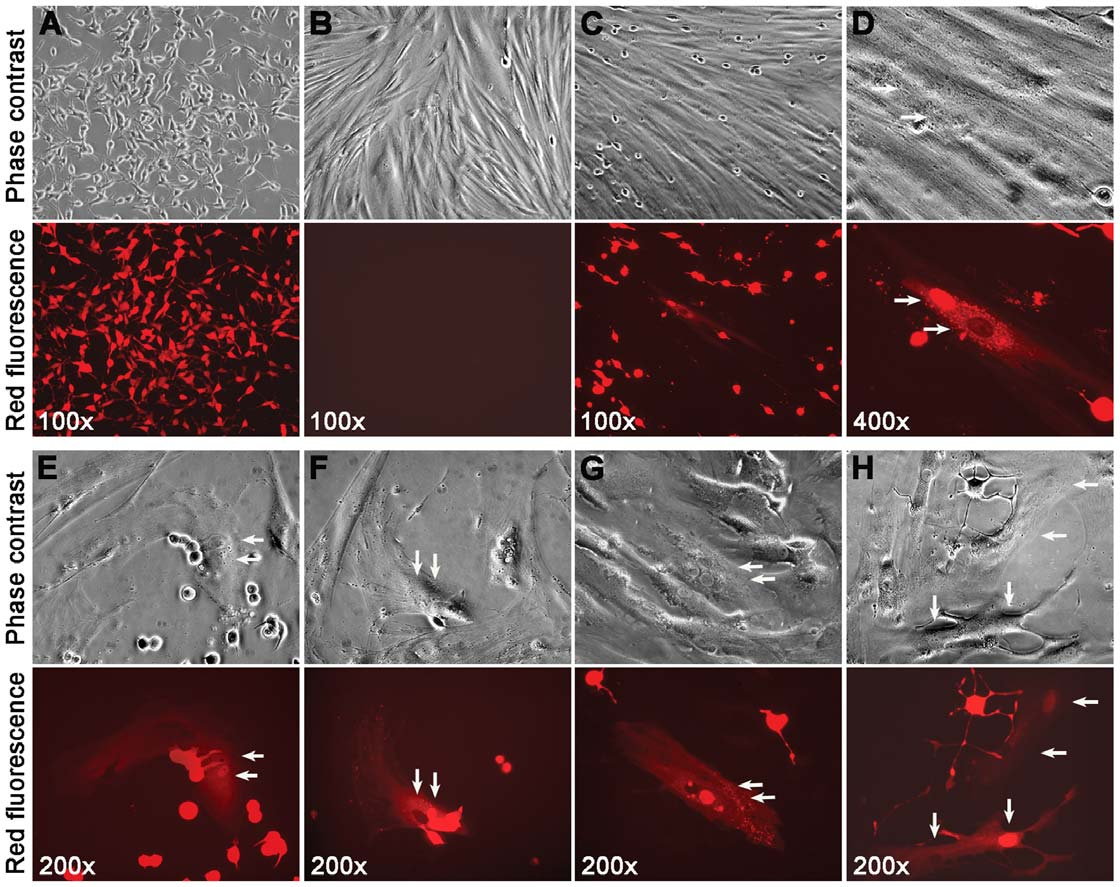
Characteristics of the spontaneous cancer-stromal cell fusion. RL-1 cells (**A**) and HPS-15 cells (**B**) are shown in separate culture. After 7 days of co-culture, spontaneous fusion could be seen (**C**). At higher magnification, the fused cell contained two nuclei, one fluorescently red and the other fluorescently pale (**D**). Cancer-stromal fusion was frequently seen in areas where RL-1 and HPS-15 formed close contact (**E**). In some cases, cells in the middle of a fusion could be seen (**F**). The two nuclei could be seen close to each other (**G**) or separated (**H**). For each view, a phase contrast image (top) and red fluorescence image (bottom) are shown. Arrows are used to indicate nuclei.

To investigate cancer-stromal interaction in co-culture, RL-1 cells were overlaid onto a confluent monolayer of HPS-14 or HPS-15 cells, so the cancer cells were in direct contact with the stromal cells. RL-1 cells showed a size of about 15 µm×50 µm (Figure 2A), whereas the stromal cells were in much larger and more expanded shapes but without any red fluorescence (Figure 2B).

Daily inspection revealed that cells in the stromal monolayer could turn fluorescently red during co-culture (Figures 2C and 2D). That the red fluorescent stromal cells resulted from fusion with the RL-1 cancer cells was determined based on the following observations. First, fluorescently red stromal cells were found mostly adjacent to cancer cells (Figure 2E). Second, although real-time technology had not been used to record the dynamic process of the fusion, it was not difficult to find instances in which a cancer cell was halfway fused with a stromal cell (Figure 2F). Third, many of these stromal cells contained two nuclei, one fluorescently red indicating its derivation from a cancer cell, and the other fluorescently pale implying its stromal origin (Figures 2C to 2H). The red cells in the stromal monolayer with a stromal appearance were cytoplasmic fusion hybrids between the cancer and the stromal cells.

**Figure 3:**
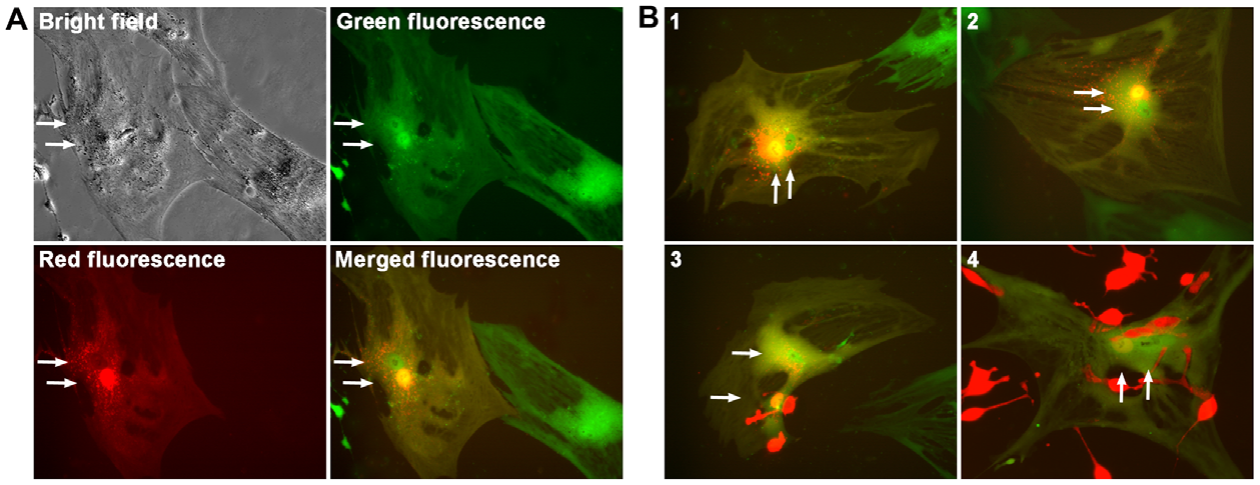
Confirming cancer-stromal cell fusion. Representative cancer-stromal cell fusion events from the co-culture of RL-1 cells with hMSC-GFP cells are shown. **A**, A single fusion event at day 7 is shown in bright field, green fluorescence, and red fluorescence. The green and red fluorescence images are merged (merged fluorescence) to show the two nuclei of different fluorescence. **B**, Merged fluorescence images for 4 additional fusion events are shown, with events 1 and 2 recorded at day 7, and 3 and 4 at day 14. Arrows are used to indicate nuclei. All the images are shown at 200× magnification.

Additional stromal cells co-cultured to observe cancer-stromal fusion. One of the studies was a co-culture of RL-1 cells with hMSC-GFP, normal human bone marrow mesenchymal stromal cells expressing a green fluorescence protein [17]. The co-culture was seen at 4 weeks with an estimated 25% population as dually fluorescent cells, green fluorescent cells with distinct stromal morphology emitting red fluorescence, most with an additional red fluorescent nucleus (Figures 3). All these studies confirmed that that the red fluorescent LNCaP prostate cancer cells were fusogenic. Once in direct contact, RL-1 cells could fuse to the stromal cells.

### 3. Characteristics of the cancer-stromal cell fusion

**Figure 4:**
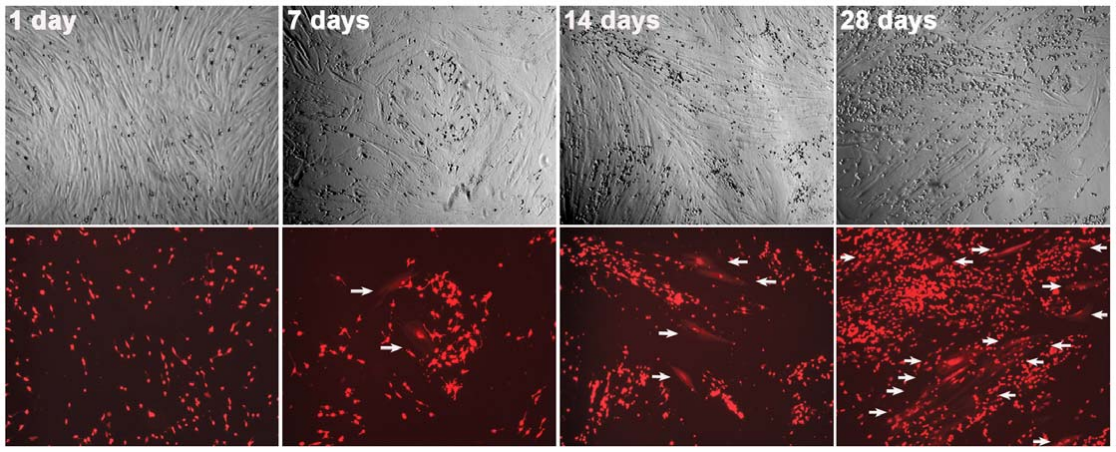
Time-dependence of cancer-stromal cell fusion. Co-cultures of RL-1 and HPS-15 cells were observed weekly for frequency of cell fusion. For each view, a phase contrast image (top) and red fluorescence image (bottom) are shown. Arrows are used to indicate cancer-stromal cell fusion events. All the images are shown at 40× magnification.

Since the stromal cell pair was slow-growing and was inhibiting the growth of RL-1 cells [11], a co-culture could be maintained for 8 weeks for periodic documentation in 4 repeated experiments. Little cell fusion was seen in the first 48 hours of co-culture. Red fluorescent cells in the stromal layer started to appear afterwards with daily increasing numbers to a plateau around 4 weeks, at which time a maximum of 20% of the stromal population was involved in the fusion (Figure 4). The cancer-stromal fusion was thus a spontaneous and a chronic process.

Cancer-stromal fusion took place only when viable cancer cells were used. Co-culture with dead RL-1 cells, killed by androgen deprivation, germicidal ultraviolet radiation, snap freezing, or vacuum desiccation, was unable to make the stromal cells fluorescently red in 8 weeks. Furthermore, incubating with purified genomic DNA from RL-1 cells (10 µg/ml) or the pAsRed2 plasmid DNA (10 µg/ml) did not cause red fluorescence in stromal cells. The spontaneous cancer-stromal fusion seemed an active process, requiring participation of both the cancer and the stromal cells.

In the 4 separate co-culture experiments, we did not find a difference between the normal HPS-14 and the cancer-associated HPS-15 stromal cells in terms of their frequency of fusion with cancer cells. RL-1 could also fuse with other matched pairs of human prostate stromal cells established in our laboratory [11] and with stromal cells derived from other tissues, including bone marrow mesenchymal stromal cells (Figure 3). The fusogenic cancer cells seemed to have the potential to fuse with a wide array of other cells.

In the co-culture with prostate stromal cells, we noticed that hybrid cells rarely yielded progenies that retained the stromal morphology. By following 32 hybrid cells from day 7 to day 28 in a co-culture, for instance, we did not find any of the hybrids dividing into clonal clusters with stromal morphology, nor from the co-culture with the hMSC-GFP cells (Figure 3). The hybrids seemed to have an interesting fate: they either had difficulty initiating cell division, or their offspring had acquired divergent morphology.

### 4. Fate of the cancer-stromal hybrids

Cell fusion is a biological process serving essential functions [22], [23], [24], [25]. While cytoplasmic fusion *per se* is a mechanism of synergism, it also provides an opportunity for nuclear fusion, leading to production of daughter cells heterogeneous from both parents. We tracked the fate of the cancer-stromal hybrid cells to assess the pathologic significance. RL-1 cells were co-cultured with HPS-14 and HPS-15 cells for 4 weeks, and were then treated with G418 (200 µg/ml) for 7 days to remove stromal cells not involved in the fusion. RL-1 cells attaching to these stromal cells were removed simultaneously. The enriched hybrid cells were collected in single-cell suspension, plated by limiting dilution, and subjected to colony formation for another 8 weeks.

**Figure 5:**
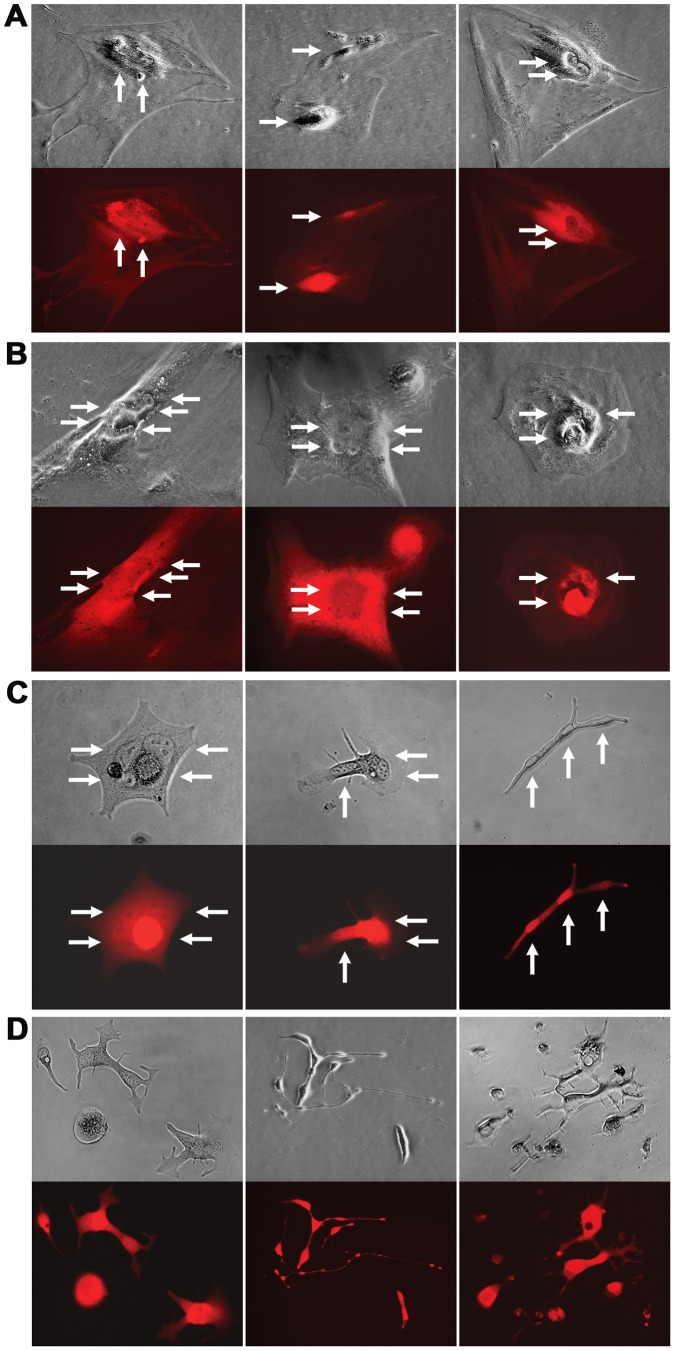
Tracking the fate of cancer-stromal hybrids. Representative morphologies of the hybrid cells during colony formation are shown. **A**, Two weeks into the culture, most of the hybrids contained two nuclei of similar fluorescence. No cell division was observed. **B**, Four weeks into the co-culture, hybrid cells adopted atypical morphology with multiple nuclei. No cell division was observed. **C**, Six weeks into the culture, the remaining hybrid cells became thin or narrow, with multiple nuclei in segments of the cell. **D**, Eight weeks into the culture, cell division became prevalent. The cell division was abnormal because it produced daughter cells in varied shapes and with reduced viability. For each view, a phase contrast image (top) and red fluorescence image (bottom) are shown. When necessary, arrows are used to indicate nuclei. All the images are shown at 200× magnification.

The hybrids shared morphologic and behavioral features throughout the colony formation assay. At the beginning, singular hybrids exhibited dramatically expanded sizes, most containing two nuclei, both showing similar red fluorescence (Figure 5A). A hybrid could survive for more than 4 weeks under the assay conditions, agreeing with the marked survival potential of parental stromal cells [11]. Remarkably, hybrid cells appeared quiescent, since no cell division was seen during the first 4 weeks of colony formation. In comparison, the parental RL-1 and stromal cells by this time formed substantial colonies in substantial sizes in parallel control assays. After 4 weeks of colony formation, many of the hybrid cells adopted atypical morphologies and accumulated additional nuclei (Figure 5B). The inability of dividing seemed to be due to deficiencies in cytokinesis rather than in DNA replication.

About half of the hybrids perished 6 weeks into colony formation. Although the cause of cell death has yet to be defined, it is known that fusion between cells with differential rates of proliferation leads to mitotic catastrophe [26], [27]. Inside a cancer-stromal hybrid, the conflicting control of mitosis by the two asynchronized nuclei could result either in a failure in the initiation of cell division, or in an abnormal mitosis and asymmetric division. A cell in mitotic catastrophe dies through genetically programmed mechanisms [28]. The cause of the hybrid cell death was likely mitotic catastrophe.

The remaining cells at this time became shrunken, displaying an elongated shape, containing multiple nuclei in different segments of the cell (Figure 5C). None of these cells survived to grow into colonies. Notably, cell division did seem to be initiated in less than 5% of the remaining hybrids, because plural cells started to appear in a few culture wells.

The cell division only became conspicuous at 8 weeks. Given that only a small fraction of hybrids survived to this time, cell division was prevalent. The division, however, appeared abnormal because the daughter cells showed mutually divergent morphologies (Figure 5D), and most died eventually. Based on these observations and by tracking 2,800 hybrids in 4 repeated studies (Table 1), we concluded that the principal fate of the cancer-stromal hybrids was death. Prostate stromal cells, after being fused by cancer cells, could function as a barrier against the survival of the cancer cells.

**Table 1:** Reduced colony formation in cancer-stromal fusion hybrids.

Cells tested	Single cells plated^a^	Colonies formed^b^	Efficiency (%)
RL-1	141^c^	14	9.9
HPS-14	125^c^	54	43.2
HPS-15	132^c^	69	52.3
RL-1/HPS-14 hybrids	1456^d^	0	0
RL-1/HPS-15 hybrids	1441^d^	41	2.8

a Wells containing a single cell 24 hours after the plating were enumerated.

b Colonies from the wells containing a single cell were enumerated.

c Data were from one colony formation assay.

d Data were combined results from 4 repeated colony formation assays.

**Figure 6:**
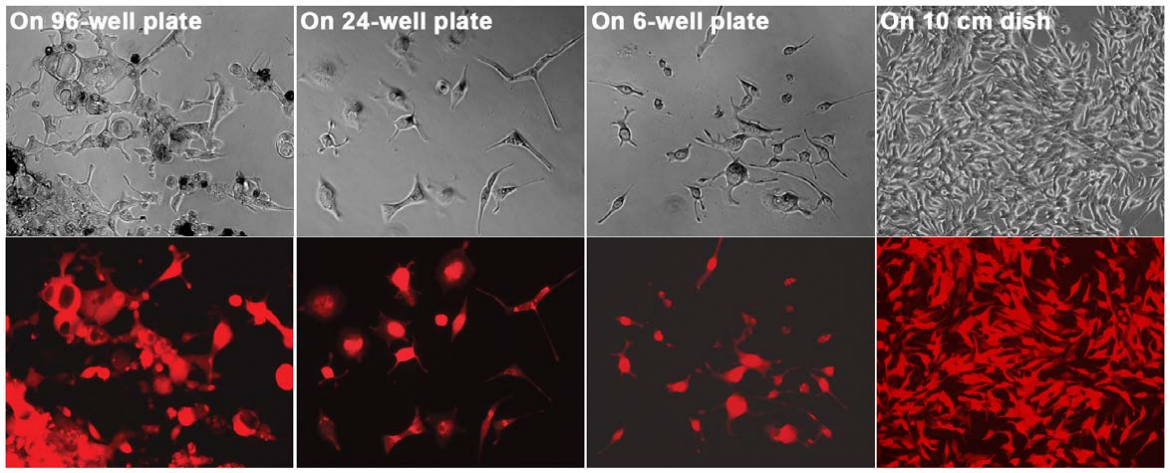
Morphologic changes in the derivative clones during colony formation. The morphology of a derivative colony was followed during the cloning process, growing from a single well of a 96-well plate to a single well of 24-well plate, to a single well of a 6-well plate, and to a 10 cm dish. For each view, a phase contrast image (top) and red fluorescence image (bottom) are shown. All the images are shown at 100× magnification.

Compared to normal counterpart, the barrier function in cancer-associated HPS-15 stromal cells could be deficient, because some hybrids from the fusion with HPS-15 survived and grew into colonies. From 4 studies of RL-1 and HPS-15 co-culture, we were able to establish 9, 6, 12, and 14 clones from each study, respectively (Table 1). The morphology and growth of the derivative cells changed along the cloning process. In general, cells with plural nuclei would disappear, those with atypical morphology would perish, cell sizes would decrease, and the growth rate would increase. By the time the clones grew into a 1×10^6^ population, which took about 20 divisions, the cells in a derivative clone would appear in a uniform morphology that was almost indistinguishable from the parental cancer cells (Figure 6). It seemed that the immediate offspring of cancer-stromal hybrids were unstable. The instability, however, was gradually lost during the proliferation process.

### 5. Androgen independence of the derivative clones from cancer-stromal fusion

**Figure 7:**
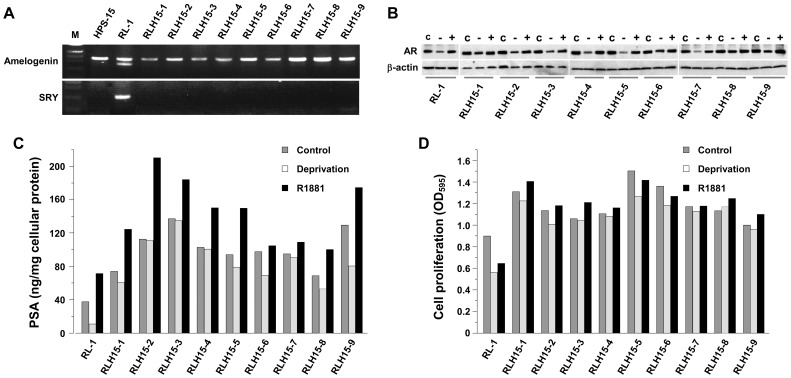
Genotypic and phenotypic changes in the derivative clones from cancer-stromal fusion. Genotypic and phenotypic parameters of the first 9 clones of the RL-1 and HPS-15 fusion hybrids were compared to those of the first 12 clones from control cloning. Compared to RL-1 clones, all the derivative clones lost Y chromosomes (**A** versus **B**). Detected by Western blotting, some of the derivative clones showed persistent AR expression even under androgen-deprivaton (**C** versus **D**). In these studies, cells were cultured for 48 hours in regular culture medium (C), androgen deprivation medium (−), and androgen deprivation medium containing 5 nM R1881 (+). The derivative clones were detected to express increased levels of PSA, even during androgen-deprivation (**E** versus **F**). When growth rate was assayed by MTT conversion, clones derived from cancer-stromal fusion displayed accelerated growth in androgen-independent fashion (**G** versus **H**). Data represent the mean of triplicate assays. For all the data points, standard deviation was less than 5% of the mean and is not shown.

We characterized the first 9 derivative clones obtained from RL-1 fusion with the HPS-15 cells (Table 1), which were named from RLH15-1 to RLH15-9. At the genomic level, all clones lost completely the Y chromosomes (Figure 7A). Instead of the XXYY karyotype known to the parental RL-1 cells, these clones bore an XX karyotype similar to that of the androgen-independent C4-2 derivative cells in the LNCaP lineage [21]. Although these clones expressed similar levels of AR protein to the parental RL-1 cells, a few (*i.e.*, clones 1, 6, 8, and 9) displayed sustained AR levels during androgen deprivation (Figure 7B), suggestive of androgen insensitivity. Importantly, all these clones expressed markedly elevated levels of PSA, most of which was barely reduced upon androgen deprivation (Figure 7C), indicating androgen independence. In cell proliferation assays, all the clones showed significantly increased growth rates compared to the parental RL-1 cells, while androgen deprivation only partially inhibited the growth of these clones (Figure 7D). Increased AR level, PSA production and proliferation rate, together with insensitivity to androgen deprivation, are generally associated with malignant status. It seemed that the derivative clones had acquired additional malignant traits.

A similar study was conducted with the single-cell RL-1 clones isolated from a parallel control colony formation assay (Table 1). The first 12 clones were examined. None of these clones lost Y chromosomes (Figure 7E), and no sustained AR level was detected upon androgen deprivation (Figure 7F). All these clones displayed androgen-dependent PSA production (Figure 7G) and growth (Figure 7H). The results from this control study suggested that the development of androgen independence in the derivative clones from cancer-stromal hybrids was the result of cancer-stromal fusion, rather than clonal deviation in the RL-1 cells *per se*.

## Discussions

This study exposed spontaneous fusion between prostate cancer and stromal cells (Figures 1, 2, and 3), and uncovered fusogenecity and its spontaneity as inherent characteristics of the cancer cells. Whereas this report describes the spontaneous fusion between a single RL-1 clone and one pair of prostate stromal cells, we have observed frequent cancer-stromal fusion from the co-culture of 5 additional red fluorescent LNCaP clones with 3 matched pairs of prostate stromal cell lines that were established and characterized previously [11]; while red fluorescent clones of other commonly used prostate cancer cell lines (*e.g.*, C4-2, C4-2B, PC-3, and DU145) could all fuse with these stromal cells (data not shown). As cancer and the surrounding stromal cells have previously been shown to interact through humoral routes, these cells are now found to be able to engage a more direct and intimate relationship. Importantly, the documented high frequency (Figure 4) implies that the pathologic relevance of cancer-stromal fusion is not trivial: a similar scenario may exist *in vivo* where cancer cells can acquire adjacency to stromal cells in the tumor microenvironment.

By assessing the consequence, we found that most cancer-stromal hybrids had difficulty initiating proper cell division. Most died, probably due to mitotic catastrophe (Figure 5). This finding could be indicative of a protective role of stromal compartment in forestalling cancer spreading. Nonetheless, a small fraction of the hybrids might survive (Table 1 and Figure 6), and the surviving clones displayed genomic alteration together with signs of androgen-independence (Figure 7). Cancer-stromal fusion plays dichotomic roles in cancer progression.

### 1. Implications of the cancer-stromal cell fusion

Somatic cell fusion is one of the original theories of cancer etiology [29]. Several types of cancer cell are shown previously to be fusogenic. Melanoma cells, for example, fuse with macrophages [30], [31], while breast cancer cells fuse with endothelial cells [32]. Cell fusion is a stepwise process. Cytoplasmic fusion would result in a hybrid with segregated nuclei containing the respective parental genomes. In some hybrid cells, nuclear fusion could occur following a successful mitosis, producing daughter cells of an admixed genome [22], [33]. The derivative cells display varied inheritance of phenotypic features from both parents, detected as increased tumor cell heterogeneity [34], [35], [36], [37], [38], [39], [40]. In regard to pathologic consequence, fusion was found in some studies to induce growth arrest and cell death [38], [39], while in others it yielded derivative cells with increased malignant potential [34], [36], [41].

Spontaneity of the fusion argues that the fate of the cancer-stromal hybrids is consequential to the course of prostate cancer progression. While death by mitotic catastrophe can purge tumor infiltration, survival of the hybrids may lead to genomic reprogramming conferring stromal properties to derivative cancer cells. Most cancer-stromal hybrid cells in colony formation assays were committed to growth arrest and cell death (Figure 5), in agreement with earlier reports that cell fusion suppresses malignancy [39]. On the other hand, a few did survive to grow into derivative clones with acquired malignancy (Table 1 and Figures 6 and 7), in alignment with that cell fusion promoted tumor progression [35], [36], [41]. We thus observed contrasting effects from cell fusion within a single experimental setting.

### 2. Cancer-stromal fusion as a mechanism for stromal cells to prevent cancer progression

Mitotic catastrophe in the hybrid cells is a function of the differential growth between parental cells [26], [42], [43]. Upon cytoplasmic fusion, cell division in the hybrid would be controlled by two asynchronized nuclei. As the parental cancer and stromal cells have quite different proliferation rates (Figure 1A), the hybrid will either fail to initiate a cell division, or proceed to an abnormal division to produce offspring with reduced viability. Unless additional survival factors are available, these fragile offspring would be eliminated by cell death mechanisms.

By demonstrating the annihilation of cancer cells through fusion, this study implicates prostate stromal cells as inhibitors of cancer growth and survival. After fusing with cancer cells, most of the stromal cells became latent (Figure 5). After a long latency, the involved stromal cells perished, together with the cancer cells. The results from colony formation assays (Table 1) showed that more than 97% of the hybrids would perish, suggesting that the death of the fused cells is an efficient strategy for eliminating cancer cells. The stromal compartment may be an innate defense system against tumor spreading.

A limitation of this protective strategy is that the stromal cell has to sacrifice itself in order to eliminate the fusing cancer cell. During the chronic process of prostate cancer progression, it is possible to lose large numbers of stromal cells due to cancer-stromal fusion. Our preliminary results suggested that compared to the androgen-dependent LNCaP cells, highly aggressive and bone metastatic prostate cancer cells were more fusogenic, causing much severe stromal cell loss (data not shown). Significance of stromal cell loss caused by cancer-stromal fusion should be assessed as a mechanism for waste syndrome and cancer cachexia.

### 3. Cancer-stromal fusion as a mechanism for stromal cells to promote cancer progression

This study also revealed that cancer cells fused with the cancer-associated stromal cells could escape stromal annihilation (Table 1). Although only a small fraction of fused cells survived, the surviving cells acquired additional malignant properties (Figure 7). Remarkably, cell fusion may be the source of the acquired malignancy, since the clones from the control assay remained androgen-dependent (Figure 7). This finding is in concordance of our previous findings that co-inoculation with stromal cells *in vivo* could promote androgen independent progression of the otherwise androgen-dependent LNCaP prostate cancer cells [9], [10], and that co-culture with stromal cells *in vitro* could rescue the LNCaP cells from androgen-deprivation [11], [12], [13].

Although the matched pair of normal HPS-14 and cancer-associated HPS-15 stromal cells were equally susceptible to fusion by RL-1 cells, only hybrids from the HPS-15 fusion survived to form derivative clones in repeated experiments (Table 1), albeit at a very low frequency. The leaky stromal annihilation may be an explanation for the appearance of tumor dormancy and recurrence, while cancer-associated stromal cells seem to have a differential tropic effect. Investigations will be conducted to identify the tumor-promoting factors active in cancer-associated stromal cells.

It is intriguing that whereas the parental RL-1 cells contain Y chromosomes (Figure 1D), all 9 derivative clones from cancer-stromal fusion completely lost this chromosome (Figure 7). Y chromosome loss is seen in cancer-associated HPS-15 stromal cells (Figure 1D), and is common in aged males [44], [45] as well as in tumor cells [46]. In the LNCaP lineage, while the androgen-dependent LNCaP cells show an XXYY genotype, majorities of the androgen-independent derivative sublines have lost specifically the Y chromosome [21]. Although its pathologic significance remains obscure, loss of the Y chromosome in the 9 derivative clones could be a sign of genomic instability or a consequence of genomic reorganization. Cancer-stromal co-culture provides an experimental model for studying Y chromosomal loss.

### 4. Cancer-stromal fusion as a therapeutic target

Cell fusion is a stepwise process, initiated at direct contact and mediated by proteins on apposed cytoplasmic membranes [22], [33]. Cytoplasmic fusion among trophoblasts, myocytes, or osteoclasts leads to functional synchrony and synergism, whereas cytoplasmic fusion between different cell lineages causes cellular heterogeneity. Most critically, cytoplasmic fusion places nonaffiliated nuclei in vicinity in a single cell, a condition for nuclear fusion. During fertilization, nuclear fusion is followed by genomic reorganization and recombination, leading to the formation of a unique genome divergent from both parents. Nuclear fusion between an antibody-producing B lymphocyte and a myeloma cell results in the formation of hybridoma, an antibody-producing cell with acquired immortality. The cancer-stromal cell fusion observed in this study may follow similar steps, first cytoplasmic fusion and then nuclear fusion, followed by genomic reorganization between the aneuploid cancer and the euploid stromal cells to yield hybrid offspring inheriting varied phenotypic features of parental cells. Cancer-stromal fusion could bring forth the appearance of acquired cancer cell heterogeneity, the driving force for cancer progression and for the co-evolution between cancer and stromal cells [12], [13]. Cancer-stromal cell fusion is thus a potential therapeutic target for prostate cancer progression.

A few surface proteins are known to mediate cell fusion [25], [47]. Proteins of the human endogenous retroviral gene family and cognate receptors are known fusogens [22], [33], while tetraspanin proteins are active players both in cell fusion and in cancer progression. Abnormal expression of these proteins is found in several human cancers [22], [33]. Both expressional suppression and structural interference can experimentally compromise the function of fusogens and prevent cell fusion [48], [49]. It will be intriguing to evaluate the efficacy of these strategies for the inhibition of prostate cancer progression and metastasis. Molecular identification of the proteins mediating cancer-stromal cell fusion in prostate cancer is vital.

## References

[1] Arnold JT, Isaacs JT (2002) Mechanisms involved in the progression of androgen-independent prostate cancers: it is not only the cancer cell's fault. Endocr Relat Cancer 9: 61–73. 11914183 10.1677/erc.0.0090061 PMC4124629

[2] Navarro D, Luzardo OP, Fernandez L, Chesa N, Diaz-Chico BN (2002) Transition to androgen-independence in prostate cancer. J Steroid Biochem Mol Biol 81: 191–201. 12163131 10.1016/s0960-0760(02)00064-x

[3] Taplin ME, Balk SP (2004) Androgen receptor: a key molecule in the progression of prostate cancer to hormone independence. J Cell Biochem 91: 483–490. 14755679 10.1002/jcb.10653

[4] Chaudhary KS, Abel PD, Lalani EN (1999) Role of the Bcl-2 gene family in prostate cancer progression and its implications for therapeutic intervention. Environ Health Perspect 107 Suppl 149–57. 10229706 10.1289/ehp.99107s149 PMC1566368

[5] Chung LW, Baseman A, Assikis V, Zhau HE (2005) Molecular insights into prostate cancer progression: the missing link of tumor microenvironment. J Urol 173: 10–20. 15592017 10.1097/01.ju.0000141582.15218.10

[6] Chung LW (1993) Implications of stromal-epithelial interaction in human prostate cancer growth, progression and differentiation. Semin Cancer Biol 4: 183–192. 8318694

[7] Gleave M, Hsieh JT, Gao CA, von Eschenbach AC, Chung LW (1991) Acceleration of human prostate cancer growth in vivo by factors produced by prostate and bone fibroblasts. Cancer Res 51: 3753–3761. 1712249

[8] Gleave ME, Hsieh JT, von Eschenbach AC, Chung LW (1992) Prostate and bone fibroblasts induce human prostate cancer growth in vivo: implications for bidirectional tumor-stromal cell interaction in prostate carcinoma growth and metastasis. J Urol 147: 1151–1159. 1372662 10.1016/s0022-5347(17)37506-7

[9] Wu HC, Hsieh JT, Gleave ME, Brown NM, Pathak S, et al (1994) Derivation of androgen-independent human LNCaP prostatic cancer cell sublines: role of bone stromal cells. Int J Cancer 57: 406–412. 8169003 10.1002/ijc.2910570319

[10] Thalmann GN, Anezinis PE, Chang SM, Zhau HE, Kim EE, et al (1994) Androgen-independent cancer progression and bone metastasis in the LNCaP model of human prostate cancer. Cancer Res 54: 2577–2581. 8168083

[11] Sun X, He H, Xie Z, Qian W, Zhau HE, et al (2010) Matched pairs of human prostate stromal cells display differential tropic effects on LNCaP prostate cancer cells. In Vitro Cell Dev Biol Anim 46: 538–546. 20383666 10.1007/s11626-010-9309-z PMC2875468

[12] Rhee HW, Zhau HE, Pathak S, Multani AS, Pennanen S, et al (2001) Permanent phenotypic and genotypic changes of prostate cancer cells cultured in a three-dimensional rotating-wall vessel. In Vitro Cell Dev Biol Anim 37: 127–140. 11370803 10.1290/1071-2690(2001)037<0127:PPAGCO>2.0.CO;2

[13] Sung SY, Hsieh CL, Law A, Zhau HE, Pathak S, et al (2008) Coevolution of prostate cancer and bone stroma in three-dimensional coculture: implications for cancer growth and metastasis. Cancer Res 68: 9996–10003. 19047182 10.1158/0008-5472.CAN-08-2492 PMC3105756

[14] Lieber MM (1992) DNA ploidy: early malignant lesions. J Cell Biochem Suppl 16H 44–46. 10.1002/jcb.240501210 1289672

[15] Peters JM, Crissman JD (1989) Histopathologic diagnosis and classification of prostate adenocarcinoma: biologic significance. Henry Ford Hosp Med J 37: 8–13. 2670842

[16] Chung LW, Gleave ME, Hsieh JT, Hong SJ, Zhau HE (1991) Reciprocal mesenchymal-epithelial interaction affecting prostate tumour growth and hormonal responsiveness. Cancer Surv 11: 91–121. 1726790

[17] Spees JL, Olson SD, Whitney MJ, Prockop DJ (2006) Mitochondrial transfer between cells can rescue aerobic respiration. Proc Natl Acad Sci U S A 103: 1283–1288. 16432190 10.1073/pnas.0510511103 PMC1345715

[18] Wang R, Xu J, Saramaki O, Visakorpi T, Sutherland WM, et al (2004) PrLZ, a novel prostate-specific and androgen-responsive gene of the TPD52 family, amplified in chromosome 8q21.1 and overexpressed in human prostate cancer. Cancer Res 64: 1589–1594. 14996714 10.1158/0008-5472.can-03-3331

[19] Hasegaw T, Sato F, Ishida N, Fukushima Y, Mukoyama H (2000) Sex determination by simultaneous amplification of equine SRY and amelogenin genes. J Vet Med Sci 62: 1109–1110. 11073085 10.1292/jvms.62.1109

[20] Eng B, Ainsworth P, Waye JS (1994) Anomalous migration of PCR products using nondenaturing polyacrylamide gel electrophoresis: the amelogenin sex-typing system. J Forensic Sci 39: 1356–1359. 7815018

[21] Murillo H, Schmidt LJ, Karter M, Hafner KA, Kondo Y, et al (2006) Prostate cancer cells use genetic and epigenetic mechanisms for progression to androgen independence. Genes Chromosomes Cancer 45: 702–716. 16615098 10.1002/gcc.20333

[22] Larsson LI, Bjerregaard B, Talts JF (2008) Cell fusions in mammals. Histochem Cell Biol 129: 551–561. 18351375 10.1007/s00418-008-0411-1 PMC2323033

[23] Spees JL, Olson SD, Ylostalo J, Lynch PJ, Smith J, et al (2003) Differentiation, cell fusion, and nuclear fusion during ex vivo repair of epithelium by human adult stem cells from bone marrow stroma. Proc Natl Acad Sci U S A 100: 2397–2402. 12606728 10.1073/pnas.0437997100 PMC151352

[24] Chen EH, Grote E, Mohler W, Vignery A (2007) Cell-cell fusion. FEBS Lett 581: 2181–2193. 17395182 10.1016/j.febslet.2007.03.033

[25] Podbilewicz B (2006) Cell fusion. WormBook 1–32. 10.1895/wormbook.1.52.1 PMC4781059 18050486

[26] Castedo M, Perfettini JL, Roumier T, Andreau K, Medema R, et al (2004) Cell death by mitotic catastrophe: a molecular definition. Oncogene 23: 2825–2837. 15077146 10.1038/sj.onc.1207528

[27] Castedo M, Perfettini JL, Roumier T, Yakushijin K, Horne D, et al (2004) The cell cycle checkpoint kinase Chk2 is a negative regulator of mitotic catastrophe. Oncogene 23: 4353–4361. 15048074 10.1038/sj.onc.1207573

[28] Castedo M, Perfettini JL, Roumier T, Valent A, Raslova H, et al (2004) Mitotic catastrophe constitutes a special case of apoptosis whose suppression entails aneuploidy. Oncogene 23: 4362–4370. 15048075 10.1038/sj.onc.1207572

[29] Aichel O (1911) Über Zellverschmelzung mit qualitative abnormer chromosomenverteilung. In: W R, editor. Vorträge und Aufsätze über Entwicklungsmechanik der Organismen. Leipzig: Wilhelm Engelmann. 1–115.

[30] Chakraborty AK, Sodi S, Rachkovsky M, Kolesnikova N, Platt JT, et al (2000) A spontaneous murine melanoma lung metastasis comprised of host x tumor hybrids. Cancer Res 60: 2512–2519. 10811133

[31] Rachkovsky M, Sodi S, Chakraborty A, Avissar Y, Bolognia J, et al (1998) Melanoma x macrophage hybrids with enhanced metastatic potential. Clin Exp Metastasis 16: 299–312. 9626809 10.1023/a:1006557228604

[32] Mortensen K, Lichtenberg J, Thomsen PD, Larsson LI (2004) Spontaneous fusion between cancer cells and endothelial cells. Cell Mol Life Sci 61: 2125–2131. 15316661 10.1007/s00018-004-4200-2 PMC11138582

[33] Larsson LI, Bjerregaard B, Wulf-Andersen L, Talts JF (2007) Syncytin and cancer cell fusions. ScientificWorldJournal 7: 1193–1197. 17704852 10.1100/tsw.2007.212 PMC5900956

[34] Barski G, Cornefert F (1962) Characteristics of “hybrid”-type clonal cell lines obtained from mixed cultures in vitro. J Natl Cancer Inst 28: 801–821. 13865344

[35] Barski G, Sorieul S, Cornefert F (1961) “Hybrid” type cells in combined cultures of two different mammalian cell strains. J Natl Cancer Inst 26: 1269–1291. 13687369

[36] Busund LT, Killie MK, Bartnes K, Olsen R, Seljelid R (2002) Spontaneous hybridization of macrophages and Meth A sarcoma cells. Int J Cancer 98: 573–581. 11920618 10.1002/ijc.10249

[37] Wakeling WF, Greetham J, Bennett DC (1994) Efficient spontaneous fusion between some co-cultured cells, especially murine melanoma cells. Cell Biol Int 18: 207–210. 8019495 10.1006/cbir.1994.1063

[38] Wiener F, Dalianis T, Klein G, Harris H (1974) Cytogenetic studies on the mechanism of formation of isoantigenic variants in somatic cell hybrids. I. Banding analyses of isoantigenic variant sublines derived from the fusion of TA3Ha carcinoma with MSWBS sarcoma cells. J Natl Cancer Inst 52: 1779–1796. 4834408 10.1093/jnci/52.6.1779

[39] Wiener F, Fenyo EM, Klein G (1974) Tumor-host cell hybrids in radiochimeras. Proc Natl Acad Sci U S A 71: 148–152. 4521047 10.1073/pnas.71.1.148 PMC387954

[40] Pawelek JM (2005) Tumour-cell fusion as a source of myeloid traits in cancer. Lancet Oncol 6: 988–993. 16321767 10.1016/S1470-2045(05)70466-6

[41] Pawelek JM (2000) Tumour cell hybridization and metastasis revisited. Melanoma Res 10: 507–514. 11198471 10.1097/00008390-200012000-00001

[42] Canman CE (2001) Replication checkpoint: preventing mitotic catastrophe. Curr Biol 11: R121–124. 11250164 10.1016/s0960-9822(01)00057-4

[43] King KL, Cidlowski JA (1995) Cell cycle and apoptosis: common pathways to life and death. J Cell Biochem 58: 175–180. 7673325 10.1002/jcb.240580206

[44] Stone JF, Sandberg AA (1995) Sex chromosome aneuploidy and aging. Mutat Res 338: 107–113. 7565866 10.1016/0921-8734(95)00016-y

[45] Wong AK, Fang B, Zhang L, Guo X, Lee S, et al (2008) Loss of the Y chromosome: an age-related or clonal phenomenon in acute myelogenous leukemia/myelodysplastic syndrome? Arch Pathol Lab Med 132: 1329–1332. 18684036 10.5858/2008-132-1329-LOTYCA

[46] Verma RS, Manikal M, Conte RA, Godec CJ (1999) Chromosomal basis of adenocarcinoma of the prostate. Cancer Invest 17: 441–447. 10434955 10.3109/07357909909021436

[47] Oren-Suissa M, Podbilewicz B (2007) Cell fusion during development. Trends Cell Biol 17: 537–546. 17981036 10.1016/j.tcb.2007.09.004

[48] Bjerregaard B, Holck S, Christensen IJ, Larsson LI (2006) Syncytin is involved in breast cancer-endothelial cell fusions. Cell Mol Life Sci 63: 1906–1911. 16871371 10.1007/s00018-006-6201-9 PMC11136146

[49] Vargas A, Moreau J, Landry S, LeBellego F, Toufaily C, et al (2009) Syncytin-2 plays an important role in the fusion of human trophoblast cells. J Mol Biol 392: 301–318. 19616006 10.1016/j.jmb.2009.07.025

